# (*E*)-4-[(4-Amino-5-bromo­pyridin-3-yl)­iminometh­yl]phenol

**DOI:** 10.1107/S160053680800812X

**Published:** 2008-05-10

**Authors:** Mingjian Cai, Mingjie Zhang, Yongliang Hu

**Affiliations:** a Department of Chemistry, Tianjin University, Tianjin 300072, People’s Republic of China

## Abstract

In the mol­ecule of the title compound, C_12_H_10_BrN_3_O, the pyridine and benzene rings are oriented at a dihedral angle of 34.93 (3)°. Intra­molecular N—H⋯N and N—H⋯Br hydrogen bonds result in the formation of two non-planar five-membered rings. In the crystal structure, inter­molecular O—H⋯N and N—H⋯O hydrogen bonds link the mol­ecules to form a three-dimensional network.

## Related literature

For general background, see: Liu *et al.* (2002 ▶).
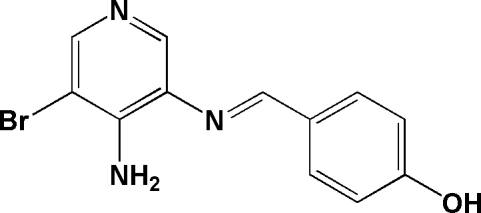


## Experimental

### 

#### Crystal data

C_12_H_10_BrN_3_O
*M* *_r_* = 292.14
Monoclinic, 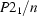

*a* = 4.9607 (10) Å
*b* = 15.586 (3) Å
*c* = 14.906 (3) Å
β = 95.65 (3)°
*V* = 1146.9 (4) Å^3^
*Z* = 4
Mo *K*α radiation
μ = 3.57 mm^−1^
*T* = 113 (2) K
0.10 × 0.08 × 0.06 mm

#### Data collection

Rigaku Saturn diffractometer
Absorption correction: multi-scan (Blessing, 1995 ▶) *T*_min_ = 0.717, *T*_max_ = 0.814
14191 measured reflections
2739 independent reflections
2123 reflections with *I* > 2σ(*I*)
*R*_int_ = 0.053

#### Refinement

*R*[*F* ^2^ > 2σ(*F* ^2^)] = 0.040
*wR*(*F* ^2^) = 0.100
*S* = 1.02
2739 reflections
163 parameters
2 restraints
H atoms treated by a mixture of independent and constrained refinement
Δρ_max_ = 0.53 e Å^−3^
Δρ_min_ = −0.59 e Å^−3^

### 

Data collection: *CrystalClear* (Rigaku/MSC, 2005 ▶); cell refinement: *CrystalClear*; data reduction: *CrystalClear*; program(s) used to solve structure: *SHELXS97* (Sheldrick, 2008 ▶); program(s) used to refine structure: *SHELXL97* (Sheldrick, 2008 ▶); molecular graphics: *SHELXTL* (Sheldrick, 2008 ▶); software used to prepare material for publication: *SHELXTL* and *PLATON* (Spek, 2003 ▶).

## Supplementary Material

Crystal structure: contains datablocks global, I. DOI: 10.1107/S160053680800812X/hk2438sup1.cif

Structure factors: contains datablocks I. DOI: 10.1107/S160053680800812X/hk2438Isup2.hkl

Additional supplementary materials:  crystallographic information; 3D view; checkCIF report

## Figures and Tables

**Table 1:** Hydrogen-bond geometry (Å, °)

*D*—H⋯*A*	*D*—H	H⋯*A*	*D*⋯*A*	*D*—H⋯*A*
O1—H1⋯N1^i^	0.82	1.88	2.688 (3)	167
N3—H3*A*⋯Br1	0.877 (18)	2.72 (3)	3.125 (4)	110.0 (19)
N3—H3*A*⋯O1^ii^	0.877 (18)	2.54 (2)	2.967 (5)	111.0 (19)
N3—H3*B*⋯N2	0.889 (16)	2.28 (3)	2.700 (5)	109 (2)

Symmetry codes: (i) 
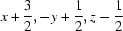
; (ii) 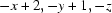.

**Fig. 1.**
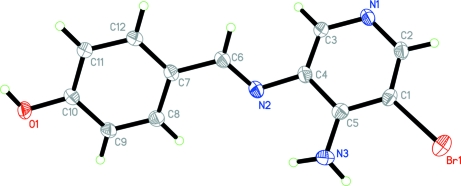
The molecular structure of the title molecule, with the atom-numbering scheme. Displacement ellipsoids are drawn at the 35% probability level.

**Fig. 2.**
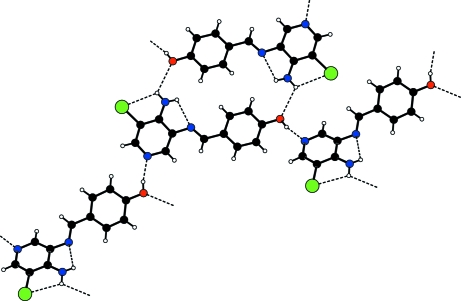
A partial packing diagram of (I). Hydrogen bonds are shown as dashed lines.

## supplementary crystallographic information

### Comment

Schiff bases, as substrates, are important organic intermediates. Their recent applications in asymmetric catalytic hydrogenation, asymmetric chemical reduction and oxidation and asymmetric alkylidation of carbon atom, as well as reactions with Lawessen regent are very active (Liu *et al.*, 2002). We have recently synthesized the novel title compound, (I), and report herein its crystal structure.

In the molecule of the title compound, (I), (Fig. 1) the bond lengths and angles are within normal ranges. Rings A (N1/C1–C5) and B (C7–C12) are, of course, planar and the dihedral angle between them is A/B = 34.93 (3)°. The intramolecular N—H···N and N—H···Br hydrogen bonds (Table 1) result in the formation of two non-planar five-membered rings; C (N2/C4/C5/N3/H3B) and D (Br1/C1/C5/N3/H3A).

In the crystal structure, intermolecular O—H···N and N—H···O hydrogen bonds (Table 1) link the molecules to form a three-dimensional network (Fig. 2), in which they may be effective in the stabilization of the structure.

### Experimental

5-bromopyridine-3,4-diamine (1.88 g, 10 mmol) was added to a solution of 4-hydroxybenzaldehyde (1.22 g, 10 mmol)in MeOH (50 ml). The solution was refluxed for 10 h, and then dried over magnesium sulfate, filtered and the volatiles were removed under reduced pressure. The crude product was further purified and recystallized from MeOH affording yellow crystals of (I) (yield; 70%).

### Refinement

H atoms (for NH_2_) were located in a difference synthesis and refined isotropically [N—H = 0.876 (10) and 0.889 (10) Å; U_iso_(H) = 0.028 (8) and 0.027 (8) Å^2^]. The remaining H atoms were positioned geometrically, with O—H = 0.82 Å (for OH) and C—H = 0.93 Å for aromatic H, respectively, and constrained to ride on their parent atoms with U_iso_(H) = xU_eq_(C,O), where x = 1.5 for OH H and x = 1.2 for aromatic H atoms.

### Crystal data

**Table:**

C_12_H_10_BrN_3_O	*F*_000_ = 584
*M**_r_* = 292.14	*D*_x_ = 1.692 Mg m^−^^3^
Monoclinic, *P*2_1_/*n*	Mo *K*α radiation λ = 0.71073 Å
Hall symbol: -P 2yn	Cell parameters from 3259 reflections
*a* = 4.9607 (10) Å	θ = 1.9–27.8º
*b* = 15.586 (3) Å	µ = 3.57 mm^−^^1^
*c* = 14.906 (3) Å	*T* = 113 (2) K
β = 95.65 (3)º	Plate, yellow
*V* = 1146.9 (4) Å^3^	0.10 × 0.08 × 0.06 mm
*Z* = 4	

### Data collection

**Table:**

Rigaku Saturn diffractometer	2739 independent reflections
Radiation source: rotating anode	2123 reflections with *I* > 2σ(*I*)
Monochromator: confocal	*R*_int_ = 0.053
*T* = 113(2) K	θ_max_ = 27.9º
ω scans	θ_min_ = 2.6º
Absorption correction: multi-scan(Blessing, 1995)	*h* = −6→6
*T*_min_ = 0.717, *T*_max_ = 0.814	*k* = −20→20
14191 measured reflections	*l* = −19→19

### Refinement

**Table:**

Refinement on *F*^2^	Secondary atom site location: difference Fourier map
Least-squares matrix: full	Hydrogen site location: inferred from neighbouring sites
*R*[*F*^2^ > 2σ(*F*^2^)] = 0.040	H atoms treated by a mixture of independent and constrained refinement
*wR*(*F*^2^) = 0.100	*w* = 1/[σ^2^(*F*_o_^2^) + (0.0558*P*)^2^ + 0.0115*P*] where *P* = (*F*_o_^2^ + 2*F*_c_^2^)/3
*S* = 1.02	(Δ/σ)_max_ = 0.001
2739 reflections	Δρ_max_ = 0.53 e Å^−^^3^
163 parameters	Δρ_min_ = −0.59 e Å^−^^3^
2 restraints	Extinction correction: none
Primary atom site location: structure-invariant direct methods	

### Special details

**Table:**

Geometry. All e.s.d.'s (except the e.s.d. in the dihedral angle between two l.s. planes) are estimated using the full covariance matrix. The cell e.s.d.'s are taken into account individually in the estimation of e.s.d.'s in distances, angles and torsion angles; correlations between e.s.d.'s in cell parameters are only used when they are defined by crystal symmetry. An approximate (isotropic) treatment of cell e.s.d.'s is used for estimating e.s.d.'s involving l.s. planes.
Refinement. Refinement of F^2^ against ALL reflections. The weighted R-factor wR and goodness of fit S are based on F^2^, conventional R-factors R are based on F, with F set to zero for negative F^2^. The threshold expression of F^2^ > 2sigma(F^2^) is used only for calculating R-factors(gt) etc. and is not relevant to the choice of reflections for refinement. R-factors based on F^2^ are statistically about twice as large as those based on F, and R- factors based on ALL data will be even larger.

### Fractional atomic coordinates and isotropic or equivalent isotropic displacement parameters (Å^2^)

**Table:**

	*x*	*y*	*z*	*U*_iso_*/*U*_eq_	
Br1	0.22060 (6)	0.579793 (19)	0.40491 (2)	0.03830 (14)	
O1	1.2134 (4)	0.29970 (11)	−0.18529 (12)	0.0258 (4)	
H1	1.2791	0.2515	−0.1871	0.039*	
N1	−0.0149 (4)	0.34708 (13)	0.29398 (14)	0.0207 (5)	
N2	0.4754 (4)	0.40060 (14)	0.12855 (15)	0.0214 (5)	
N3	0.5400 (5)	0.53733 (15)	0.23987 (17)	0.0263 (5)	
C1	0.1953 (5)	0.48149 (16)	0.32940 (17)	0.0213 (5)	
C2	0.0159 (5)	0.41727 (16)	0.34483 (18)	0.0220 (6)	
H2	−0.0886	0.4231	0.3930	0.026*	
C3	0.1392 (5)	0.34101 (15)	0.22483 (17)	0.0197 (5)	
H3	0.1206	0.2921	0.1890	0.024*	
C4	0.3231 (5)	0.40235 (16)	0.20351 (17)	0.0199 (5)	
C5	0.3553 (5)	0.47740 (16)	0.25818 (17)	0.0202 (5)	
C6	0.5619 (5)	0.32877 (16)	0.10100 (17)	0.0209 (5)	
H6	0.5200	0.2789	0.1310	0.025*	
C7	0.7226 (5)	0.32179 (16)	0.02482 (17)	0.0192 (5)	
C8	0.7946 (5)	0.39343 (16)	−0.02422 (18)	0.0224 (6)	
H8	0.7318	0.4474	−0.0098	0.027*	
C9	0.9575 (5)	0.38505 (16)	−0.09363 (18)	0.0232 (6)	
H9	1.0021	0.4333	−0.1257	0.028*	
C10	1.0566 (5)	0.30420 (16)	−0.11623 (17)	0.0202 (5)	
C11	0.9849 (5)	0.23290 (16)	−0.06839 (17)	0.0208 (5)	
H11	1.0475	0.1789	−0.0829	0.025*	
C12	0.8202 (5)	0.24182 (16)	0.00099 (17)	0.0213 (6)	
H12	0.7736	0.1933	0.0324	0.026*	
H3A	0.508 (6)	0.5891 (10)	0.2593 (19)	0.028 (8)*	
H3B	0.590 (5)	0.5330 (19)	0.1844 (9)	0.027 (8)*	

### Atomic displacement parameters (Å^2^)

**Table:**

	*U*^11^	*U*^22^	*U*^33^	*U*^12^	*U*^13^	*U*^23^
Br1	0.0515 (2)	0.02764 (19)	0.0375 (2)	−0.00060 (13)	0.01289 (16)	−0.01098 (13)
O1	0.0335 (11)	0.0214 (9)	0.0253 (10)	0.0049 (8)	0.0168 (8)	0.0036 (8)
N1	0.0210 (11)	0.0234 (11)	0.0186 (11)	0.0013 (9)	0.0061 (9)	0.0025 (9)
N2	0.0192 (11)	0.0245 (11)	0.0212 (12)	0.0001 (9)	0.0060 (9)	0.0012 (9)
N3	0.0292 (13)	0.0188 (12)	0.0321 (14)	−0.0044 (10)	0.0090 (11)	−0.0002 (10)
C1	0.0241 (13)	0.0191 (12)	0.0207 (13)	0.0038 (10)	0.0024 (11)	−0.0032 (10)
C2	0.0235 (14)	0.0238 (13)	0.0195 (13)	0.0042 (11)	0.0067 (11)	0.0009 (11)
C3	0.0220 (13)	0.0181 (12)	0.0194 (13)	0.0009 (10)	0.0038 (10)	−0.0005 (10)
C4	0.0211 (13)	0.0219 (12)	0.0171 (13)	0.0029 (10)	0.0040 (10)	0.0014 (10)
C5	0.0205 (13)	0.0185 (12)	0.0211 (13)	0.0032 (10)	0.0003 (10)	0.0015 (10)
C6	0.0187 (13)	0.0227 (13)	0.0216 (13)	−0.0001 (10)	0.0042 (10)	0.0021 (11)
C7	0.0181 (12)	0.0229 (13)	0.0168 (13)	−0.0019 (10)	0.0030 (10)	0.0001 (10)
C8	0.0235 (13)	0.0191 (12)	0.0253 (14)	0.0027 (11)	0.0063 (11)	0.0031 (11)
C9	0.0269 (14)	0.0180 (12)	0.0259 (14)	0.0019 (11)	0.0093 (11)	0.0058 (11)
C10	0.0196 (13)	0.0223 (12)	0.0193 (13)	−0.0008 (10)	0.0039 (10)	0.0022 (10)
C11	0.0222 (13)	0.0186 (12)	0.0222 (13)	−0.0003 (10)	0.0054 (11)	−0.0001 (10)
C12	0.0228 (13)	0.0187 (12)	0.0229 (14)	−0.0013 (10)	0.0059 (11)	0.0025 (10)

### Geometric parameters (Å, °)

**Table:**

Br1—C1	1.898 (2)	C3—H3	0.9300
O1—C10	1.352 (3)	C4—C5	1.426 (3)
O1—H1	0.8200	C6—C7	1.455 (3)
N1—C2	1.331 (3)	C6—H6	0.9300
N1—C3	1.346 (3)	C7—C12	1.396 (3)
N2—C6	1.281 (3)	C7—C8	1.400 (3)
N2—C4	1.409 (3)	C8—C9	1.380 (4)
N3—C5	1.355 (3)	C8—H8	0.9300
N3—H3A	0.876 (10)	C9—C10	1.406 (3)
N3—H3B	0.889 (10)	C9—H9	0.9300
C1—C2	1.374 (4)	C10—C11	1.386 (3)
C1—C5	1.388 (4)	C11—C12	1.387 (3)
C2—H2	0.9300	C11—H11	0.9300
C3—C4	1.380 (3)	C12—H12	0.9300
			
C10—O1—H1	109.5	N2—C6—C7	122.8 (2)
C2—N1—C3	116.9 (2)	N2—C6—H6	118.6
C6—N2—C4	119.6 (2)	C7—C6—H6	118.6
C5—N3—H3A	115 (2)	C12—C7—C8	117.8 (2)
C5—N3—H3B	113.1 (19)	C12—C7—C6	119.7 (2)
H3A—N3—H3B	117 (3)	C8—C7—C6	122.4 (2)
C2—C1—C5	121.5 (2)	C9—C8—C7	120.9 (2)
C2—C1—Br1	119.7 (2)	C9—C8—H8	119.5
C5—C1—Br1	118.76 (19)	C7—C8—H8	119.5
N1—C2—C1	122.9 (2)	C8—C9—C10	120.6 (2)
N1—C2—H2	118.5	C8—C9—H9	119.7
C1—C2—H2	118.5	C10—C9—H9	119.7
N1—C3—C4	124.5 (2)	O1—C10—C11	123.1 (2)
N1—C3—H3	117.8	O1—C10—C9	118.0 (2)
C4—C3—H3	117.8	C11—C10—C9	118.8 (2)
C3—C4—N2	126.0 (2)	C10—C11—C12	120.2 (2)
C3—C4—C5	118.4 (2)	C10—C11—H11	119.9
N2—C4—C5	115.5 (2)	C12—C11—H11	119.9
N3—C5—C1	124.8 (2)	C11—C12—C7	121.6 (2)
N3—C5—C4	119.4 (2)	C11—C12—H12	119.2
C1—C5—C4	115.8 (2)	C7—C12—H12	119.2
			
C3—N1—C2—C1	−0.2 (4)	N2—C4—C5—C1	176.8 (2)
C5—C1—C2—N1	0.7 (4)	C4—N2—C6—C7	−179.3 (2)
Br1—C1—C2—N1	179.2 (2)	N2—C6—C7—C12	177.2 (2)
C2—N1—C3—C4	−0.4 (4)	N2—C6—C7—C8	−0.1 (4)
N1—C3—C4—N2	−175.8 (2)	C12—C7—C8—C9	−0.2 (4)
N1—C3—C4—C5	0.4 (4)	C6—C7—C8—C9	177.1 (2)
C6—N2—C4—C3	−35.7 (4)	C7—C8—C9—C10	−0.5 (4)
C6—N2—C4—C5	148.0 (2)	C8—C9—C10—O1	179.5 (2)
C2—C1—C5—N3	−178.7 (2)	C8—C9—C10—C11	0.8 (4)
Br1—C1—C5—N3	2.9 (3)	O1—C10—C11—C12	−179.1 (2)
C2—C1—C5—C4	−0.7 (4)	C9—C10—C11—C12	−0.6 (4)
Br1—C1—C5—C4	−179.13 (17)	C10—C11—C12—C7	−0.1 (4)
C3—C4—C5—N3	178.2 (2)	C8—C7—C12—C11	0.5 (4)
N2—C4—C5—N3	−5.1 (3)	C6—C7—C12—C11	−176.9 (2)
C3—C4—C5—C1	0.2 (3)		

### Hydrogen-bond geometry (Å, °)

**Table:**

*D*—H···*A*	*D*—H	H···*A*	*D*···*A*	*D*—H···*A*
O1—H1···N1^i^	0.82	1.88	2.688 (3)	167
N3—H3A···Br1	0.877 (18)	2.72 (3)	3.125 (4)	110.0 (19)
N3—H3A···O1^ii^	0.877 (18)	2.54 (2)	2.967 (5)	111.0 (19)
N3—H3B···N2	0.889 (16)	2.28 (3)	2.700 (5)	109 (2)

Symmetry codes: (i) *x*+3/2, −*y*+1/2, *z*−1/2; (ii) −*x*+2, −*y*+1, −*z*.
